# Immunosignature Differentiation of Non-Infectious Meningoencephalomyelitis and Intracranial Neoplasia in Dogs

**DOI:** 10.3389/fvets.2018.00097

**Published:** 2018-05-11

**Authors:** Bathilda B. Lake, John Henry Rossmeisl, Julie Cecere, Phillip Stafford, Kurt L. Zimmerman

**Affiliations:** ^1^Department of Biomedical Sciences and Pathobiology, Virginia-Maryland College of Veterinary Medicine, Blacksburg, VA, United States; ^2^Department of Small Animal Clinical Sciences, Virginia-Maryland College of Veterinary Medicine, Blacksburg, VA, United States; ^3^Biodesign Institute, Center for Innovations in Medicine, Arizona State University, Tempe, AZ, United States

**Keywords:** meningoencephalomyelitis of unknown etiology, intracranial neoplasia, immunosignature, peptide microarray, canine

## Abstract

A variety of inflammatory conditions of unknown cause (meningoencephalomyelitis of unknown etiology—MUE) and neoplastic diseases can affect the central nervous system (CNS) of dogs. MUE can mimic intracranial neoplasia both clinically, radiologically and even in some cases, histologically. Serum immunosignature protein microarray assays have been used in humans to identify CNS diseases such as Alzheimer’s and neoplasia, and in dogs, to detect lymphoma and its progression. This study evaluated the effectiveness of immunosignature profiles for distinguishing between three cohorts of dogs: healthy, intracranial neoplasia, and MUE. Using the learned peptide patterns for these three cohorts, classification prediction was evaluated for the same groups using a 10-fold cross validation methodology. Accuracy for classification was 100%, as well as 100% specific and 100% sensitive. This pilot study demonstrates that immunosignature profiles may help serve as a minimally invasive tool to distinguish between MUE and intracranial neoplasia in dogs.

## Introduction

A variety of infectious, noninfectious, vascular, demyelinating and neoplastic disorders of the central nervous system (CNS) are recognized in dogs. (1–4) Serology and PCR remain effective tools in humans and canines for screening many of the infectious causes. (5–8) For many of the remaining non-neoplastic disorders, underlying inflammation associated with an immunologic disturbance is thought to be the basis for their pathogenesis. (2, 3) Often the initiating cause of the inflammation remains unknown and these cases are referred to as meningoencephalomyelitis of unknown etiology (MUE). (2, 3) MUE has been classically described as a disease that occurs in several types of small purebred dogs, but atypical forms have been identified in a variety of dog breeds. (9) These atypical MUE presentations are the most problematic to the diagnostician. Human studies prove that atypical noninfectious inflammatory conditions can mimic intracranial neoplasia both clinically, radiologically and even histologically. (10) Further complicating the matter is that treatment optimization and long-term prognosis can differ for these two different classes of conditions (inflammation / neoplasia). (1, 2, 11) Tissue biopsy for histopathology is the definitive method used to distinguish between these disorders, but it is rarely performed due to associated financial cost and patient risk. (1, 4, 12) What is needed is a reliable, easily replicated, non-invasive method that can distinguish between MUE and intracranial neoplasia in dogs.

Towards this goal, blood immunosignature peptide profiles have shown great promise in distinguishing between various disease states in human studies. (13–15) Immunosignaturing is not limited to use in a single species or for a single disorder and can be performed using a single blood sample. (13) The methodology is dependent upon the host’s immunologic response pattern to proteins uniquely expressed in association with the disease of interest. (16, 17) Immunosignature randomly displays the circulating antibodies on a machine-readable peptide microarray chip allowing for an unbiased display of all types of antibody binding. (18) The peptides on the microarray are randomly generated and take advantage of the cross-reactivity of patient antibodies to epitopes on the peptide array. (18) In addition, the arrays are inexpensive and have the potential to be used for high throughput sample processing. (18) The power of immunosignaturing lies in its ability to learn about a disease through accumulation of patient data. Immunosignaturing works only after training with patients who have known diseases so that machine learning algorithms can identify underlying protein binding patterns in the microarray chip. These learned expression patterns are then used to classify new patients into the disease categories used for training. High degree of accuracy (97%, *n* = 42) has already been shown using this method in dogs to distinguish between normal and those affected with lymphoma, as well as subtypes of lymphoma, and even relapse time following therapy. (18) Others have shown that, in humans, this approach can distinguish between Alzheimer’s disease, CNS tumor type, grade and healthy control patients. (14, 15, 19, 20) In another study involving 1,600 subjects, the array distinguished between 14 different diseases with 92% accuracy. (15) In this study, we evaluated the capability of the immunosignature to differentiate MUE from intracranial neoplasia in dogs by measuring its sensitivity, specificity and accuracy.

## Materials and Methods

### Study Plan

This was a retrospective pilot-study that used banked serum from 38 client owned dogs. The dogs presented for intracranial disease or as healthy dogs and were placed into one of three cohorts: MUE, intracranial neoplasia, and healthy. Cases originated from the Virginia Maryland College of Veterinary Medicine, Veterinary Teaching Hospital (VTH) or affiliated regional referral specialty practices between 1/8/2008- 10/7/2015. Client consent was obtained for use of these samples and the study was approved by the Institutional Animal Care and Use Committee of Virginia Tech.

Inclusion criteria for all the dogs were: 1–15 years old, any breed and any age. Healthy dogs were recruited from the patient population presenting for yearly pre-breeding wellness exams and had a normal wellness exam, complete blood cell count and biochemistry profile. The weight range for the healthy dogs was 10–20 kg with an average of 12 kg. The criteria for inclusion in the neoplastic group were clinical signs of brain disease and histopathological diagnosis of intracranial neoplasia by a board certified anatomic veterinary pathologist. Criteria for inclusion in the MUE group included examination by a board-certified neurologist, clinical signs of intracranial disease, brain MRI findings compatible with described MUE variants including granulomatous meningoencephalitis (GME), necrotizing meningoencephalitis (NME), or necrotizing leukoencephalitis (NLE), and albuminocytologic dissociation or pleocytosis documented on CSF analysis.(21–23) Dogs with clinicopathologic or imaging features displaying overlap between GME, NME, and NLE were assigned diagnoses of MUE.(3). In addition, all dogs in the MUE cohort had negative infectious disease testing results. Infectious disease testing performed in all MUE dogs included evaluation of serum antibody titers against toxoplasmosis (ELISA IgG/IgM), neosporosis (indirect fluorescent antibody [IFA]), Ehricliha canis (IFA IgG), and rocky mountain spotted fever (IFA IgM and IgG) performed by the Infectious Disease Laboratory, Athens, GA, USA. Serum was also tested at the Infectious Disease Laboratory, Athens, GA, USA for cryptococcal antigen (latex agglutination) and urine or cerebrospinal fluid for canine distemper (RT-PCR). Urine was submitted and tested for blastomyces antigen (EIA) performed by Mira Vista Diagnostics, Indianapolis, IN, USA. Three dogs also underwent additional comprehensive PCR screening for testing of known pathogens in the following six genera: Babesia, Bartonella, Anaplasma, Ehrlichia, Rickettsia, and hemotropic Mycoplasma by Vector Borne Disease Diagnostic Laboratory, Raleigh, NC, USA. Current medications and length of clinical signs were not part of the inclusion criteria. The study cases were partitioned into two sets for use with the classification prediction algorithm: training cases and validation cases.

## Patient Sera

Serum samples were collected on all the dogs and used for the peptide microarray chip. For the healthy, intracranial neoplasia, and MUE cases, serum was separated from a 5 mL blood sample within 1 h of collection. All samples were stored at −80C. Once all samples for the study were collected, they were batch shipped frozen overnight to The Peptide Array Core at the Biodesign Institute, Arizona State University. Upon arrival the samples were kept frozen at −80C until used for microarray processing and analysis.

## Microarray

Immunosignaturing is a method by which an individual’s antibody repertoire (>108 distinct antibodies) interacts random-sequence peptides. The pattern of binding between serum antibodies and the 125,000 random peptides becomes the signature for that individual at that point in time. Most antibodies, even those that are affinity matured, can bind to noncognate targets. This binding is enhanced by the relatively dense packing of immunosignature peptides on the surface of the array.

Array Construction: Peptide microarrays are manufactured using *in situ* synthesis of 125,000 random-sequence peptides. The arrays are made from eight-inch silicon wafers that become the surface on which peptides are grown using standard BOC synthesis. Each peptide is 11.2 amino acids on average with a SD of 1.3 and a normal distribution about 11.2. Peptides are synthesized from C-terminus to N-terminus with the amine group furthest from the surface of the array and a GGG C-terminus linker.

Binding of Antibodies to the Array – Each microarray is pre-incubated in PBS pH 7.2 with 0.1% BSA for 1 h at room temperature prior to addition of serum. Serum is added to a final concentration of 1:1500 in incubation buffer. Serum is mixed by inversion for 1 h in an Agilent Hybridization oven (Agilent Inc., Santa Clara, CA) at 25°C at 20 RPM. After primary incubation, the arrays are washed in wafer, then incubation buffer, then placed in a tray where 5 ml of PBS pH 7.2 + 1% casein (Sigma, St. Louis, MO) is added. Fluorescent horse anti-canine Fc secondary antibody (ThermoFisher Scientific, Pittsburgh, PA) is added to a final concentration of 4 nM and mixed for 1 h at 25°C. Following secondary incubation, the arrays are washed 3x in incubation buffer and 3x in distilled water for 5’ each. Following the last wash, the arrays are centrifuged at 1500 g for 5’ to dry. Arrays are scanned in an Innopsys (Carbonne, France) Innoscan 910 at 1 um resolution at 647 nm excitation. 16-bit TIFF images are aligned using GenePix 6.2 software (Molecular Devices, Mountain View, CA). Raw data is analyzed using GeneSpring (Agilent Inc.) and R (CRAN Repository). Each array contains epitopes from monoclonal antibodies as controls: YPYDVPDYA which binds to anti-HA (Rockland Antibodies, Rockland, MD), AALEKDYEEVGV which binds to anti-tubulin monoclonal DM1A (Invitrogen/ThermoFisher), and TFRHSVVV which binds to anti-p53 monoclonal Ab1 (Clontech, Palo Alto, CA). Each epitope is checked for binding to the corresponding monoclonal, to ensure accurate synthesis of the array peptides.

## Statistical Analysis

TIFF images of the arrays were obtained using an Innopsys Innoscan 910 laser scanner. Raw TIFF images were obtained using a 547 nm green laser at full power, 80% PMT at 1 um resolution. 16-bit TIFF images were aligned using GenePix Pro 6.0 (Molecular Devices, Sunnyvale, CA) to produce a tab delineated results file. Data were normalized by dividing each value in an array by the median for that array. Once the first normalization was complete, the rows were normalized to the median of that row, such that the median for each row is 1. As the microarray data was log10-normal, T-test calculations were performed on log10 transformed data. Results were evaluated in GeneSpring 7.3.1 (Agilent, Santa Clara, CA). Feature selection was ANOVA post-hoc test, Tukey with FWER (Family-Wise Error Rate) =5% multiple-test correction. Cross-validation was done using at leave-one-out, leave-two-out and leave 10% out. Mean accuracy is reported. Classification is done using Support Vector Machines as implemented in GeneSpring 7.3.1. Heatmaps were generated in GeneSpring with individuals and peptides clustered using the Pearson’s Correlation for both rows (peptide) and columns (individuals). Principal component values were obtained in GeneSpring. For 3-D PCA plots, the first 3 principal components are plotted on X, Y, and Z respectively. All comparisons were adequately powered at 95% confidence, 80% power.

Algorithm accuracy, sensitivity and specificity were calculated comparing classifier predictions with true cohort membership for these samples. Preliminary immunosignature data (not shown) was used to calculate sample sizes for the pilot study. The sample sizes were calculated using the t-test-based sample size calculation in R (statistical software). Parameters for sample size calculations were: alpha = 0.05, beta = 0.80, delta = 1.3 fold minimum detectable fold-change, and standard deviations were extrapolated. (17)

## Results

There was a total of 20 dogs in the training classifier with three cohorts: 7 healthy dogs, 7 MUE dogs, and 6 intracranial neoplastic dogs (Table 1). There was a total of 18 dogs for the validation classifier with three cohorts: 7 healthy dogs, 6 MUE dogs, and 5 neoplasia dogs. A statistical summary of age, sex, and breed is listed in Table 1 for all the dogs used in the study. A summary of the specific types of intracranial neoplasia is shown in Table 2.

**Table 1:** Summary of study population signalment based on cohort.

**Class**	**Number**	**Age range**	**Sex**	**Breed**
Healthy	14	5.5 (4.9–6 years)	F	Mixed breed (14)
MUE	13	6.75 (10 mo-12 years)	MN 6FS 7	French bull dog (1), Min pin (1), Chihuahua (2), Beagle (2), Maltese mix (2), Mountain Feist (1), Boxer (2), Japanese Chin (1), Mixed breed (2)
Intracranial neoplasia	11	7.44 (5–10 years)	M 5MN 2FS 2	Boston Terrier (2), Labrador Retriever (1), Staffordshire Terrier (1), Bassett Hound (1), English Bull dog (1), Boxer (1), Mixed breed (1)

Median age is presented with the range, low to high, in parenthesis.

Meningoencephalomyelitis of unknown etiology

MN- male neutered; FS- female spayed; M- intact male; F- female intact

**Table 2:** Summary of histopathology results for intracranial neoplasia cohort.

**Type of intracranial neoplasia**	**Diagnoses, Number**
Pituitary tumor	Functional corticotroph macroadenoma, *n* = 1
Glioma	Oligodendroglioma, *n* = 4 Grade III, *n* = 3 Grade II, *n* = 1 Astrocytoma, *n* = 3 Grade IV, *n* = 2 Grade II, *n* = 1
Meningioma	Grade I, *n* = 1; Grade II, *n* = 1
Hemangiosarcoma	Metastatic from right atrium, *n* = 1

All dogs in the neoplasia and MUE cohorts had clinical signs of forebrain disease. Clinical signs were focal in 10/11 and diffuse in 1/11 dogs with intracranial neoplasia. In the MUE cohorts, neurological signs were classified by attending neurologist as multifocal in 9/13 dogs, and focal in 4/13. In 3/13 dogs with MUE, concurrent cervical spinal cord disease contributed to the multifocal neuroanatomic localization. At the time blood was collected for immune profiling, 5/13 dogs with MUE and 4/11 dogs with intracranial neoplasia had been treated with corticosteroids. No other immunosuppressive or immunomodulating medications were administered to either cohort prior to sample analyses. Based on clinical, MRI, CSF and ancillary infectious testing findings, clinical diagnoses assigned by neurologists to dogs in the MUE cohort included GME (*n* = 6), NLE (*n* = 2), NME (*N* = 1), and MUE (*n* = 4). Two dogs in the MUE group had histopathologic confirmation of disease by necropsy (*n* = 1, NLE) or stereotactic brain biopsy (*n* = 1, GME).(4)

**Figure 1:**
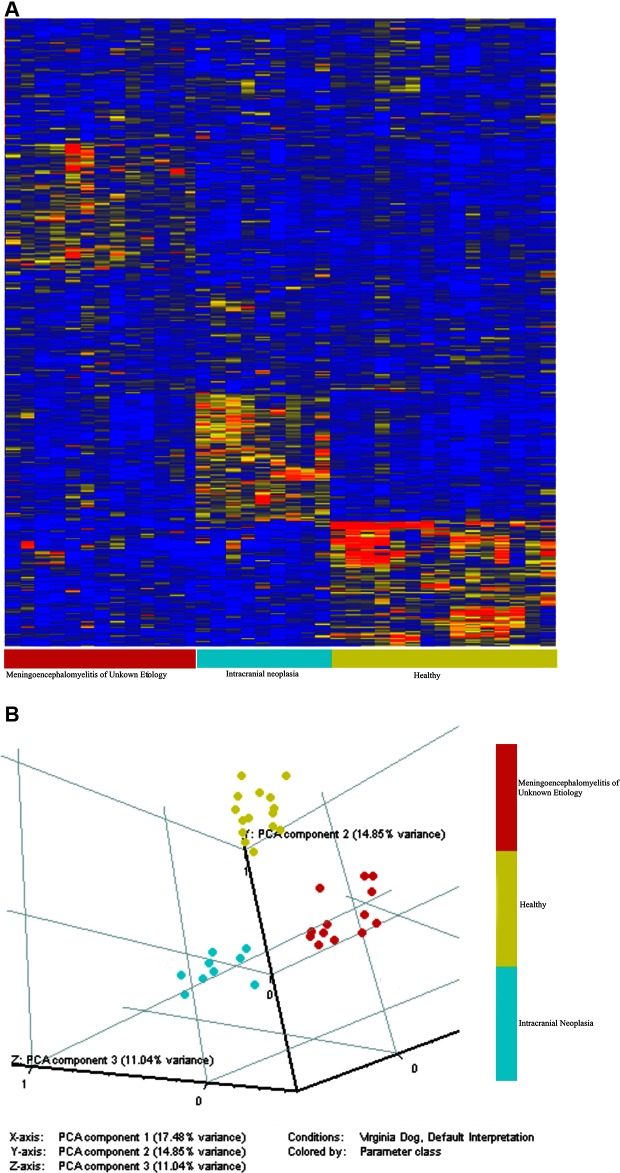
The immunosignature distinguishes healthy dogs, dogs with intracranial neoplasia, and dogs with MUE. A Student’s T-test (*p* < 0.05 with FDR) and a 1.5-fold change between classes were used to select 100 informative peptides per cohort. The distribution of intensities is shown in the Heatmap **(A)**. Colors represent the per peptide median normalized intensities. Yellow indicates the median, red fivefold above the median, and blue 0.25-fold below the median. Each row represents a peptide and each column represents and individual. Individuals were clustered in GeneSpring using the Pearson correlation to each other. Variation among individuals based on the 100 peptides is shown in the principle components graph plot **(B)**. The immunosignature distinguishes healthy dogs, dogs with intracranial neoplasia, and dogs with MUE.

A set of 100 peptides was identified for each cohort that best represented and discriminated between the cohorts, creating a total of 300 peptides. The heatmap (Figure 1A) shows the 300 peptides on the Y-axis, and the animals on the X-axis. The principal components map (Figure 1B) is 3D, and shows that the cohorts do not overlap, and the cross-validation did not misdiagnose any animals. The difference between groups on the X, Y and Z axis show that the variance across cohorts is not due to one specific feature, but rather the differences across many different features which presumes the diseases are very well predicted. There was 100% specificity, 100% sensitivity, and 100% accuracy at leave-one-out, leave-two-out and leave 10% out.

## Discussion

Immune based cancer therapies were first explored because of the well-established knowledge that cancers can generate detectable cellular and humoral immune responses. (18) This lead to the further investigation of immunosignatures to characterize diseases. In this study, immunosignature profiles were created for healthy dogs, dogs with intracranial neoplasia, and dogs with meningoencephalomyelitis of unknown etiology. These immunosignature profiles were evaluated as a potential diagnostic tool for discriminating between these three states. This limited pilot study demonstrates that immunosignature profiling has promise for differentiating between dogs with intracranial neoplasia and MUE.

Immunosignature has multiple strengths and advantages as a diagnostic tool. A previous human study looked at the resilience of immunosignature serum samples. (15) These samples that were more than 10 years old, from multiple geographical sites and from patients of varying age and sex. In spite of these factors, a robust immunosignature profile was still obtained. Immunosignature has also been used in humans to diagnosis not only several types of neoplasms, but a variety of other types of CNS and pancreatic diseases. (15) These findings suggest that a variety of disorders may have a unique immunosignature with distinctive antibody binding patterns to allow their recognition. Another strength of immunosignature is its use of serum, plasma or dried blood. (13) Once stored as a dried blood spot, samples did not break down in high heat or over time. (13) This finding strengthens the practicality of using immunosignature as a diagnostic tool in a clinical setting. Given these attributes, there is a potential for developing a database of immunosignature profiles for a variety of disease using archived serum samples.

The blood brain barrier provides the central nervous system with a protective environment limiting direct interaction with circulating blood. (24) This limiting barrier works both directions making it harder, in many cases, to use CNS analytes as blood biomarkers to identify CNS disease. Since this study uses peripheral blood samples for this very purpose, this is a potential concern. Fortunately, human studies have shown that blood immunosignature profiles can still reliably detect CNS disease. (19) This study demonstrated that healthy dogs, dogs with intracranial neoplasia, and dogs with MUE can be readily distinguished from one another using immunosignature profiles. The immunosignature classification algorithm predicted cohort membership on the independent test set with 100% specificity, 100% sensitivity, and 100% accuracy. The current cost of the assay, while not trivial, is magnitudes less than that of other diagnostics such as surgical biopsy and advanced imaging options. As this diagnostic method achieves validation and wider adoption, cost will likely decrease and further increase its use.

Although the sample size per cohort for both the validation and training phases is small, it was adequate based on power calculations. (17) A follow up prospective study could be done with more patients to help further validate this minimally invasive approach for distinguishing between the three cohorts, and more specifically differentiating between the different neoplasms. In addition, a wider variety of neoplasms should be included to further explore the value of immunosignature as a diagnostic tool.

Histopathology was used to confirm the diagnosis for the intracranial neoplasia cases; however, histopathology results were not available for many dogs in the MUE group due to cost and morbidity risk. There is a possibility that some of the MUE cases may have had an underlying neoplastic process which could confuse the training classifier algorithm leading to poor test performance in the validation cases. However, to help minimize this risk of inclusion of CNS neoplasia cases in the MUE cohort, neurological exams and imaging findings were screen by a board-certified neurologist for compatibility with MUE, which is the current standard of care for diagnosis of MUE cases.

Interestingly, the immunosignature profiles in this study also showed some distinction between the several types of intracranial neoplasia; glioma versus meningioma (data not shown). This could be an additional benefit of using immunosignatures as a diagnostic tool. Further prospective studies are required be to explore this delineation within the intracranial neoplasia group. Overall, immunosignature proved to be a reliable diagnostic tool in distinguishing between dogs without CNS signs, dogs with intracranial neoplasia, and dogs with MUE with accuracy, sensitivity, and specificity.

## Ethics Statement

This study was carried out in accordance with the recommendations of the Institutional Animal Care and Use Committee of Virginia Tech. The protocol was approved by the Institutional Animal Care and Use Committee of Virginia Tech.

## Author Contributions

BL wrote the manuscript, organized the data, and reviewed the manuscript. JR collected samples, drafted the study design, and reviewed the manuscript. JC collected the samples and reviewed the manuscript. PS processed the samples, collected the data, and reviewed the manuscript. KZ drafted the study design, supervised project, wrote the manuscript, reviewed the manuscript, and collected and organized the data.

## Conflict of Interest Statement

The authors declare that the research was conducted in the absence of any commercial or financial relationships that could be construed as a potential conflict of interest.
